# Deficiency in Galectin-3 Promotes Hepatic Injury in CDAA Diet-Induced Nonalcoholic Fatty Liver Disease

**DOI:** 10.1100/2012/959824

**Published:** 2012-04-19

**Authors:** Kazuhiro Nomoto, Takeshi Nishida, Yuko Nakanishi, Makoto Fujimoto, Ichiro Takasaki, Yoshiaki Tabuchi, Koichi Tsuneyama

**Affiliations:** ^1^Department of Diagnostic Pathology, Graduate School of Medicine and Pharmaceutical Science, University of Toyama, 2630 Sugitani, Toyama 930-0194, Japan; ^2^Department of Japanese Oriental Medicine, Graduate School of Medicine and Pharmaceutical Science, University of Toyama, 2630 Sugitani, Toyama 930-0194, Japan; ^3^Division of Molecular Genetics Research, Life Science Research Center, University of Toyama, 2630 Sugitani, Toyama 930-0194, Japan

## Abstract

Nonalcoholic fatty liver disease (NAFLD) is increasingly recognized as a condition in which excess fat accumulates in hepatocytes. Nonalcoholic steatohepatitis (NASH), a severe form of NAFLD in which inflammation and fibrosis in the liver are noted, may eventually progress to end-stage liver disease. Galectin-3, a *β*-galactoside-binding animal lectin, is a multifunctional protein. This protein is involved in inflammatory responses and carcinogenesis. We investigated whether galectin-3 is involved in the development of NASH by comparing galectin-3 knockout (gal3^−/−^) mice and wild-type (gal3^+/+^) mice with choline-deficient L-amino-acid-defined (CDAA) diet-induced NAFLD/NASH. Hepatic injury was significantly more severe in the gal3^−/−^ male mice, as compared to the gal3^+/+^ mice. Data generated by microarray analysis of gene expression suggested that galectin-3 deficiency causes alterations in the expression of various genes associated with carcinogenesis and lipid metabolism. Through canonical pathway analysis, involvement of PDGF and IL-6 signaling pathways was suggested in galectin-3 deficiency. Significant increase of CD14, Fos, and Jun, those that were related to lipopolysaccharide-mediated signaling, was candidate to promote hepatocellular damages in galectin-3 deficiency. In conclusion, galectin-3 deficiency in CDAA diet promotes NAFLD features. It may be caused by alterations in the expression profiles of various hepatic genes including lipopolysaccharide-mediated inflammation.

## 1. Introduction

Galectin-3 is a 30-kD mammalian lectin which has a C-terminal carbohydrate-recognition domain and an N-terminal domain comprising multiple repeat sequences rich in glycine, proline, and tyrosine. It is expressed in various cells such as epithelial cells and inflammatory cells. Galectin-3 performs multiple functions, including the regulation of cell-to-cell or cell-to-matrix adhesion [1–5], chemoattraction of monocytes/macrophages [6], mediation of pre-mRNA splicing [7], and protection against Fas- and staurosporine-induced apoptosis associated with Bcl-2 expression [8].

During the development of human and mouse embryos, galectin-3 is detectable in most types of cells, including hepatocytes [9, 10]. In rats, the expression of galectin-3 was found to be rapidly induced in the liver at 9 days after birth and decreased to trace levels in adults [11]. Moreover, very little galectin-3 mRNA was detected in the livers of normal adult rats [12]. In human [13] and mouse livers, galectin-3 expression was not detected in normal hepatocytes, whereas it was found to be prominent in the bile duct epithelial cells and Kupffer cells [14, 15]. On the other hand, it was found that galectin-3 expression was aberrantly induced in the cytoplasm of the periportal hepatocytes of adult rat liver exhibiting inflammation caused by CCl_4_ administration [12] and in the hepatocytes surrounding regenerating nodules in human cirrhotic liver [15]. In addition, the results of a previous study showed that the expression of galectin-3 was significantly upregulated in activated rat hepatic stellate cells [16]. Galectin-3 expression was also found to be required for the myofibroblast activation and matrix production mediated by transforming growth factor *β* (TGF-*β*) [17]. Recently, we demonstrated that disrupted galectin-3 expression in male mice leads to the development of nonalcoholic fatty liver disease (NAFLD) and hepatocellular carcinoma (HCC) with liver fibrosis [18, 19]. Taken together, these results suggest that galectin-3 plays some important roles in liver homeostasis. However, its role in liver pathology, particularly NAFLD, is largely unknown.

NAFLD is increasingly recognized as a liver disorder that may eventually progress to end-stage liver disease, including HCC [20–22]. NAFLD is the preferred term for describing a liver condition that includes a wide spectrum of conditions from simple steatosis to steatohepatitis, advanced fibrosis, and cirrhosis in the liver. The diagnostic criteria for nonalcoholic steatohepatitis (NASH) are continuously evolving and based on histological findings [23, 24]. The most common histopathological features of NASH include hepatocellular steatosis and ballooning degeneration, mixed acute and chronic lobular inflammation, and perisinusoidal fibrosis in the zone 3 regions. Although NASH can be caused by a variety of factors, its occurrence is frequently associated with obesity, type II diabetes, hyperlipidemia, and metabolic syndrome [20, 25–27]. The pathogenesis of NASH is poorly understood; however, a “two-hit theory” has been proposed [28]. This theory suggests that, in addition to steatosis (the first “hit”), certain other factor(s) (the second “hit”) are required for the development of steatohepatitis. Above all, involvement of lipopolysaccharide is broadly accepted as a candidate of trigger. Recent studies using animal models of NAFLD have also provided new insights into the molecular and physiologic alterations that contribute the first and second hits in the progression of NAFLD to end-stage liver disease [29, 30]. Various genes and their expression profiles associated with NAFLD have recently been analyzed and identified [31–34].

In the present study, we hypothesized that galectin-3 deficiency increases hepatic injury in mice with NASH induced by a choline-deficient l-amino-acid-defined (CDAA) diet, because galectin-3 is a negative regulator of lipopolysaccharide-mediated inflammation [35]. We report herein that enhancement of lipopolysaccharide signaling pathway involving *CD14*, *Fos,* and *Jun* was observed in galectin-3-deficient mice with CDAA diet. Lipopolysaccharide-mediated signaling may not only be a trigger but also be a promoter of NAFLD.

## 2. Materials and Methods

### 2.1. Animals and Experimental Protocol

Galectin-3 knockout (gal3^−/−^) mice and their wild-type littermates (gal3^+/+^) generated on a CD1 background were generously provided by Dr. Liu's group [36]. The mice were housed under pathogen-free conditions in a temperature-controlled room with a 12 h light/dark illumination cycle. All the procedures for handling animals were in accordance with the Guide for the Care and Use of Laboratory Animals and approved by the Committee on Animal Experimentation of the University of Toyama. In order to determine the maximum duration required to detect hepatic changes in mice fed the CDAA diet (Dyets, Bethlehem, PA, USA; product no. 518753), a pilot study was conducted in 8- to 10-week-old male mice fed over a period of 0–12 weeks to examine the effects of the diet on the serum alanine transaminase (ALT) and aspartate transaminase (AST) levels and on liver steatosis, inflammation, and fibrosis. The severity of liver steatosis, inflammation, and fibrosis observed after 8 weeks of CDAA diet was similar to that observed at 12 weeks; therefore, we determined that maximum duration fed CDAA diet was 8 weeks in this study. Thus, groups of 4 to 6 male gal3^−/−^ and gal3^+/+^ mice were fed the CDAA diet and sacrificed after 0, 2, 4, and 8 weeks. The body weights were recorded at the start and end of each experimental period. Blood samples were collected for serum analysis. The livers of the mice were rapidly excised, rinsed in ice-cold saline, and weighed. Liver samples were snap-frozen in liquid nitrogen and maintained at −80°C until analysis. A portion of each liver was fixed in 10% formalin for histopathological examination.

### 2.2. Evaluation of Liver Injury

The serum ALT and AST levels were measured using a transaminase CII-Test kit (Wako Pure Chemical Industries, Osaka, Japan) according to the manufacturer's protocol. Liver samples were minced in ice-cold PBS (pH 7.4) and homogenized in the buffer at a concentration of 1 : 10 (*w* : *v*). After centrifugation at 5,500 rpm for 10 min at 4°C, the supernatants were stored for subsequent determination of the total liver triglyceride levels using a commercially available kit (Wako E-test triglyceride kit; Wako Pure Chemical Industries). Data were expressed as milligrams of triglyceride per gram of wet liver weight.

### 2.3. Histological Analysis

The formalin-fixed liver tissue was processed, and 4-*μ*m-thick paraffin sections of the liver samples were stained with hematoxylin and eosin for histological analysis. Steatohepatitis was scored by experienced pathologists (K. N. and K. T.) in a blinded manner. Hepatic steatosis was graded as follows according to the percentage of lipid-laden hepatocytes: 0, 0%; 1, 0–33%; 2, 33–67%; 3, 67–100%. Necroinflammation was graded on the basis of the number and size of the necroinflammatory lesions in representative liver sections as follows: 0, none; 1, mild; 2, moderate; 3, severe [37].

### 2.4. RNA Preparation

A liver sample from one mouse in each group fed CDAA diet for 8 weeks was homogenized, and total RNA was extracted using the RNeasy Total RNA Extraction kit (Qiagen, Valencia, CA, USA) and treated with DNase I (RNase-free DNase kit; Qiagen, Valencia, CA, USA) for 15 min at room temperature to remove residual genomic DNA.

### 2.5. Affymetrix GeneChip Hybridization

In order to investigate the gene expression profiles, the Affymetrix mouse expression 430A array was used. Sample preparation for this analysis was performed according to the procedure described in the Affymetrix GeneChip Expression technical manual. Briefly, 5 *μ*g of total RNA was used to synthesize double-stranded cDNA by using a GeneChip Expression 3′-Amplification Reagents One-cycle cDNA Synthesis Kit (Affymetrix, Santa Clara, CA, USA). Biotin-labeled cRNA was then synthesized using GeneChip Expression 3′-Amplification Reagents for IVT Labeling (Affymetrix). After fragmentation, the biotinylated cRNA was hybridized to a GeneChip array at 45°C for 16 h. The chip was washed, stained with streptavidin-phycoerythrin, and scanned with a GeneChip scanner 3000 (Affymetrix), and the results were analyzed using the GeneChip Analysis Suite Software (Affymetrix). Hybridization intensity data were represented as presence/absence calls for each gene, and the differences in gene expression between experiments were detected by comparison analysis. The data were further analyzed using GeneSpring version 7.3 (Silicon Genetics, Redwood City, CA, USA) to extract significantly expressed genes and determine their ontology, including biological processes, cellular components, and molecular functions. Genes were considered upregulated or downregulated if there was 2-fold or greater difference in their expression between the gal3^−/−^ and the gal3^+/+^ mice.

### 2.6. Functional Network, Gene Ontology, and Canonical Pathway Analyses

The genes identified by GeneSpring were used for functional network and gene ontology analyses. Gene accession numbers were imported into the Ingenuity Pathway Analysis version 3.1 (IPA) software (Ingenuity Systems, Mountain View, CA, USA), and the gene products were categorized using the software on the basis of their hepatic location and cellular localization and the reported or suggested biochemical, biological, and molecular functions. The genes were then mapped to the genetic networks available in the Ingenuity database on the basis of their score-wise ranking, and the probability that a collection of genes is to the same as or greater than the number of genes in a network was a matter of chance. A score of 3 obtained randomly indicates that there is a 1/1000 chance that the identified genes are a part of the network. Therefore, scores of 3 or higher have a 99.9% confidence level of not having been generated randomly. This score was used as the cutoff for identifying gene networks.

### 2.7. Real-Time PCR

Six genes included in the highly significant canonical pathways (PDGF signaling and IL-6 signaling) were chosen for further validation by real-time PCR. Total RNA was isolated from the liver samples using Isogen reagent (Nippon Gene, Toyama, Japan). cDNA was synthesized from 400 ng of hepatic mRNA by using TaqMan Reverse Transcription Reagents (Applied Biosystems, Tokyo, Japan). PCR reactions and analyses were carried out using an Mx3000P QPCR System (Stratagene, La Jolla, CA, USA) with denaturation for 10 min at 95°C, followed by 50 PCR cycles of denaturation at 95°C for 30 s, annealing at 55°C for 1 min and extension at 72°C for 30 s. All primers and probes used for analysis were designed at Nipppon EGT (Toyama, Japan). The primer and probe sequence details are given in Table 1. The amount of mRNA was calculated using glyceraldehydes-3-phosphate dehydrogenase (*GAPDH*) as the endogenous control.

### 2.8. Statistical Analysis

The values were expressed as means ± SEM. The means were compared using the Mann-Whitney *U*-test. *P* < 0.05 was considered statistically significant.

## 3. Results

### 3.1. Effect of CDAA Diet on Liver Injury in Gal3^−/−^ Mice

Mice fed the CDAA diet showed an increase in serum ALT and AST levels and liver triglyceride levels at week 2, and the levels remained elevated up to week 8. The serum ALT and AST levels were determined to be significantly higher in the gal3^−/−^ mice, as compared to the gal3^+/+^ mice after either 2 or 8 weeks of subjection to the CDAA diet (*P* < 0.05) (Table 2), whereas the increase in the liver triglyceride levels in the gal3^−/−^ mice was not significant difference compared to the gal3^+/+^ mice.

### 3.2. Effect of CDAA Diet on Hepatic Morphology in Gal3^−/−^ Mice

**Figure 1:**
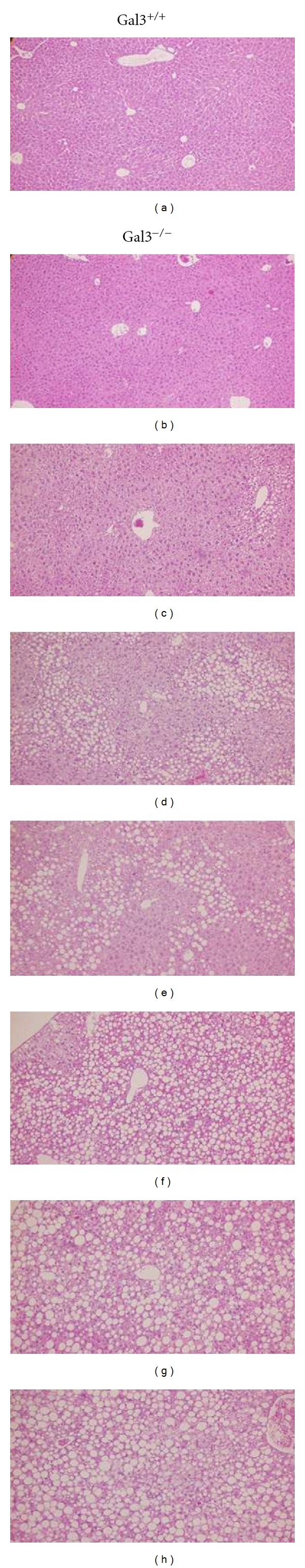
Representative photomicrographs showing the effect of choline-deficient l-amino-acid-defined (CDAA) diet on liver histology in gal3^+/+^ and gal3^−/−^ mice. (a) and (b) are week 0 of CDAA diet (Control). In both mice, histopathological differences were not observed. (c) and (d) are week 2. (e) and (f) are week 4. (g) and (h) are week 8 (hematoxylin and eosin staining, original magnification ×100).

After 2 weeks of CDAA diet consumption, macrovesicular steatosis involving lobular zones 1 and 2 was observed for both mice. The hepatocytes were swollen with mild-to-moderate micro- and macrovesicular steatosis and mild lobular and portal necroinflammation, which were more severe in the gal3^−/−^ mice (Figures 1(c) and 1(d)). In 4 weeks, steatosis was further severe in the gal3^−/−^ mice (Figures 1(e) and 1(f)). The degree of steatosis after 2 or 4 weeks was significantly higher in the gal3^−/−^ mice (*P* < 0.05) (Table 3), and the degree of necroinflammation after 2 weeks was also significantly higher in the gal3^−/−^ mice (*P* < 0.05) (Table 3). In 8 weeks, both mice showed further severe steatosis and moderate lobular and portal necroinflammation (Figures 1(g) and 1(h)).

### 3.3. Affymetrix GeneChip Analysis of Genes in CDAA Diet-Induced NAFLD

The expression of 22,625 mouse transcripts was detected using the Affymetrix mouse expression 430A array. In analysis of liver samples from the mice after 8 weeks of the CDAA diet, we identified 460 probes whose expression was upregulated by >2-fold and 185 probes whose expression was downregulated by >2-fold in gal3^−/−^ mice compared to the gal3^+/+^ mice.

### 3.4. Functional Network and Gene Ontology Analysis

To further refine our study, we investigated the biological interactions of the 645 genes identified by the GeneChip analysis using the IPA tool. We found that these 645 genes mapped to genetic networks and had functional relationships. We also identified 37 genetic networks in the livers of the gal3^−/−^ mice. Of these 37, 24 were highly significant in that they included more of the identified genes than expected by chance. Networks with high scores (>15) included more than half the identified genes (these are listed in Table 4), which are associated with cancer, cell death, cellular assembly and organization, cellular function and maintenance, organismal injury and abnormalities, carbohydrate and lipid metabolism, and so forth. We also performed gene ontology analysis using the IPA tool and identified 73 categories as significant (data not shown).

### 3.5. Canonical Pathway Analysis

**Figure 2:**
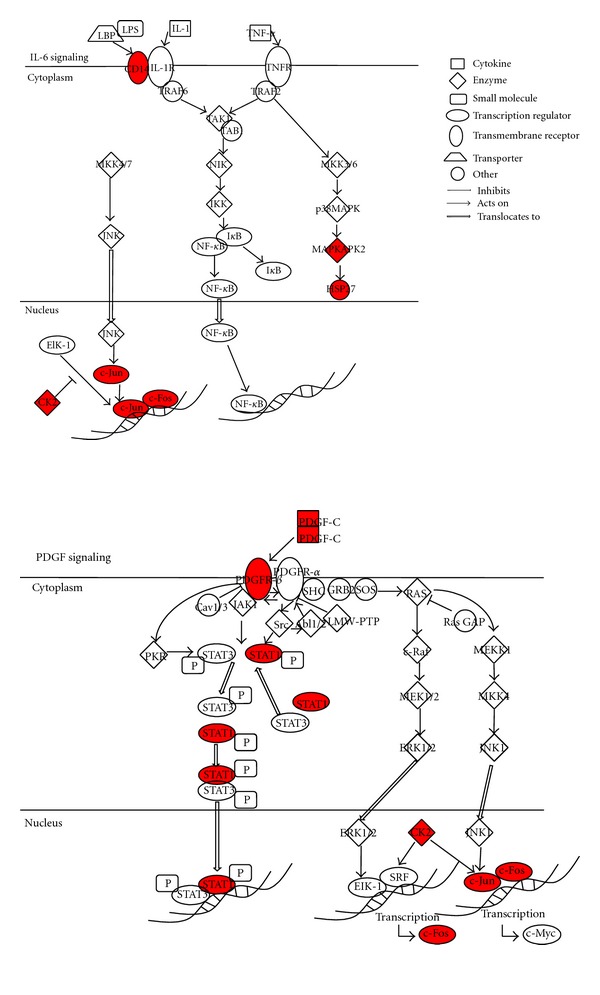
The canonical pathways of the PDGF and IL-6 signaling. These pathways were significantly identified in the complete data set. The genes indicated in red are those that were upregulated; those indicated in white are not user specified but were incorporated into the network since they shared functional relationships with the upregulated genes.

In the canonical pathway analysis, we identified 2 significant pathways (PDGF signaling and IL-6 signaling pathways) in the liver samples of gal3^−/−^ mice at after 8 weeks on the CDAA diet (Figure 2).

### 3.6. Validation of Gene Expression with Real-Time PCR

**Figure 3:**
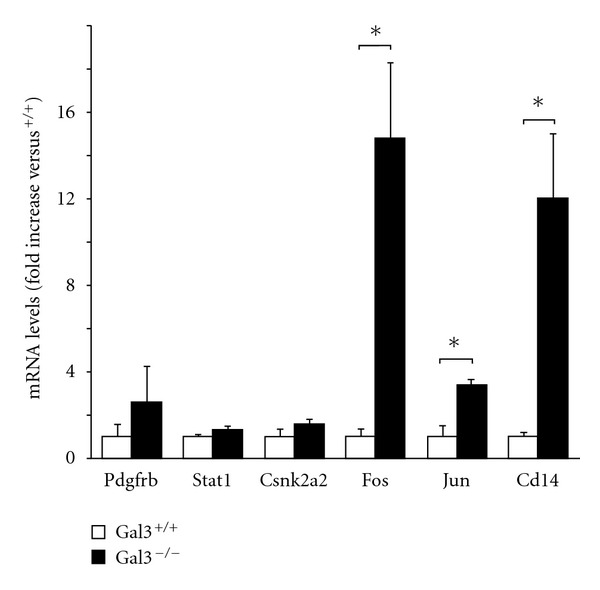
Comparison with mRNA levels of the altered genes in the significant canonical pathway (PDGF signaling and IL-6 signaling). These levels were determined by real-time PCR, normalized to glyceraldehydes-3-phosphate dehydrogenase (*GAPDH*), and expressed as fold induction relative to the gal3^+/+^ mice. The values are expressed as mean ± SEM of four mice in each group. (*P < 0.05).

In order to confirm the results of the Ingenuity pathway analysis, we measured the expression of 6 genes included in the highly significant canonical pathways. The genes in the PDGF signaling and IL-6 signaling pathway include *Pdgfrb*, *Stat1*, *Csnk2a2*, *Fos*, *Jun*, and *Cd14*. mRNAs for *Fos*, *Jun*, and *Cd14 *were significantly increased in gal3^−/−^ mice fed the CDAA diet compared to the gal3^+/+^ mice (*P* < 0.05) (Figure 3). Interestingly, these were key molecules of lipopolysaccharide-mediated inflammation. mRNAs for *Pdgfrb*, *Stat1*, and *Csnk2a2 *were also increased, but not significantly.

## 4. Discussion

Our results demonstrated that a deficiency in galectin-3 resulted in an increase in hepatic injury in CDAA diet-induced NAFLD. Additionally, the data we obtained by microarray analysis of gene expression suggested that galectin-3 deficiency, in the background of CDAA diet-induced NAFLD/NASH, may cause alterations in the expression of various genes associated with cancer, cell death, cellular assembly and organization, cellular function and maintenance, organismal injury and abnormalities, and carbohydrate and lipid metabolism. On the basis of our results, we were able to propose several potential mechanisms underlying the increase in hepatic injury observed in gal3^−/−^ mice. The first possible mechanism is an increase in hepatocyte apoptosis. Previous studies have demonstrated that hepatocyte apoptosis was significantly increased in NASH [38–40]. Furthermore, Yang et al. reported on the protective effects of galectin-3 against Fas- and staurosporine-induced apoptosis associated with Bcl-2 expression [8]. In our study, microarray analysis revealed that the expression of genes associated with cell death significantly increased in the gal3^−/−^ mice compared to the gal3^+/+^ mice. These data suggest that galectin-3 plays an important role as an antiapoptotic protein in hepatocytes affected by CDAA diet-induced NAFLD.

In the canonical pathway analysis and validation of gene expression with real-time PCR, we observed that the expression of components of the PDGF and IL-6 signaling pathways were significantly amplified in the liver of the gal3^−/−^ mice after 8 weeks of the CDAA diet. As members of the PDGF ligand family are known to play important roles in cellular proliferation and migration, this finding provided second possible mechanism behind the increased hepatic injury observed in gal3^−/−^ mice. We recently reported a case of severe fibrosis affecting multiple organs caused by neonatal PDGF overproduction [41]. In this case, the PDGF-*β* receptor was found to be overexpressed in all the fibrotic organs, including the liver. Moreover, a previous study showed that PDGF-C transgenic mice had enlarged livers associated with increased fibrosis, steatosis, cell dysplasia, and HCC [42]. These studies indicated that the overexpression of PDGF and its receptor induces a number of profibrotic pathways, suggesting that this growth factor acts as an initiator of fibrosis [42]. We speculated that galectin-3 deficiency might lead to the higher expression of genes related to the PDGF signaling pathway and consequently accelerate the development of various pathological conditions such as fibrosis and carcinogenesis in patients with NAFLD/NASH. 

In a recent landmark study, Cai et al. demonstrated that hepatic IL-6, a major proinflammatory cytokine, is markedly expressed in animal models of NAFLD [43]. Moreover, Wieckowska and coworkers found significant increase in hepatic IL-6 expression in NASH patients [44]. In the present study, the expression of genes involved in the IL-6 signaling pathway was significantly higher in the gal3^−/−^ mice. These data suggest that galectin-3 deficiency may accelerate the hepatic IL-6 signaling pathway, leading to an increased inflammatory response, steatosis, and, finally, steatohepatitis thus, providing a third possible mechanism behind the increased hepatic injury observed in gal3^−/−^ mice. Amongst related molecules of IL-6 signaling pathway, *CD14*, *Fos,* and *Jun* were significantly increased in gal3^−/−^ mice. These molecules were key molecules of lipopolysaccharide signaling. It is broadly accepted that lipopolysaccharide is one of an important trigger of NAFLD. In 8 weeks of CDAA diet, there are no significant pathological changes between gal3^−/−^ and gal3^+/+^ mice, while the titer of ALT and AST was quite high in gal3^−/−^ mice. We hypothesized that lipopolysaccharide-mediated signaling may not only be a trigger but also be a promoter of NAFLD. Galectin-3, which is a negative regulator of lipopolysaccharide-mediated inflammation, may be effective to inhibit disease promotion of NAFLD/NASH. Treatment of NAFLD by recruiting galectin-3 should be examined using animal model of NAFLD in further study. 

Previous studies have demonstrated that NASH can progress to cirrhosis and eventually to HCC [45, 46]; however, its carcinogenetic mechanisms are largely unknown. Although several animal models have been proposed in order to understand the pathogenesis of NAFLD [47], the possible neoplastic transformation that characterizes end-stage NASH has been developed in only a few models, including aged gal3^−/−^ mice [18, 48]. In the functional network and gene ontology analysis, we identified networks with high scores (>15), wherein more than half of the identified genes were associated with cancer, cell death, cellular assembly and organization, cellular function and maintenance, organismal injury and abnormalities, and carbohydrate and lipid metabolism. Therefore, we hypothesize that galectin-3 deficiency under the condition of NAFLD/NASH contributes to liver carcinogenesis.

In conclusion, our study demonstrated that a deficiency in galectin-3 increases hepatic injury in mice with CDAA diet-induced NAFLD/NASH. Recruitment of galectin-3 may be a new target of the treatment of NAFLD/NASH.

## Figures and Tables

**Table 1:** Primer pairs and probe sequences used for real-time PCR reactions.

Target	Sequences
*Pdgfrb*	F: 5′-ACCTGTTCATTTTTGTCACGGATG-3′ R: 5′-TTCTTCTCATGTAGCGTCACCTC-3′ Probe: 5′-6FAM-CGACAATTCCGTGCCGAGTGACAGACC-TAMRA-3′
*Stat 1*	F: 5′-AGGTGTTGTCAGATCGAACCTTC-3′ R: 5′-CATGCACGGCTGTCGTTCTA-3′ Probe: 5′-6FAM-CTCTTCCAGCAGCTCATTCGGAGCTC-TAMRA-3′
*Csnk2a2*	F: 5′-AGCCCGGAGGCCCTAGA-3′ R: 5′-TTTGGCGGTCAATCTCTGTTG-3′ Probe: 5′-6FAM-TCTTGACAAGCTCCTGCGGTACGACC-TAMRA-3′
*Fos*	F: 5′-CCTGAGCCCAAGCCATCC-3′ R: 5′-GTCATCAAAGGGTTCTGCCTTC-3′ Probe: 5′-6FAM-CGTTGCTGATGCTCTTGACTGGCTCCA-TAMRA-3′
*Jun*	F: 5′-ACTGCAAAGATGGAAACGACCT-3′ R: 5′-AGCCGTAGGCACCGCTCT-3′ Probe: 5′-6FAM-CGATGCCCTCAACGCCTCGTTC-TAMRA-3′
*Cd14*	F: 5′-CCGAAGCCAGATTGGTCCAG-3′ R: 5′-CACACGCTTTAGAAGGTATTCCAG-3′ Probe: 5′-6FAM-CCGCCGTACAATTCCACATCTGCCG-TAMRA-3′
*GAPDH*	F: 5′-AGGGATGATGTTCTGGGCAG-3′ R: 5′-AGACTGTGGATGGCCCCTC-3′ Probe: 5′-6FAM-ACGGCCATCACGCCACAGCTTT-TAMRA-3′

*Pdgfrb*: platelet-derived growth factor receptor, beta polypeptide; *Stat1*: signal transducer and activator of transcription 1; *Csnk2a2*: casein kinase 2, alpha prime polypeptide; *Fos*: FBJ osteosarcoma oncogene; *GAPDH*: glyceraldehyde-3-phosphate dehydrogenase.

**Table 2:** Effect of CDAA diet on serum ALT, serum AST, and liver triglyceride levels in the gal3^+/+^ and gal3^−/−^ mice.

		CDAA diet consumption			
		0 wk	2 wk	4 wk	8 wk
Serum ALT (IU/L)	Gal-3^+/+^	26.9 ± 4.3	48.2 ± 9.8	89.9 ± 33.3	64.2 ± 17.5
	Gal-3^−/−^	19.7 ± 1.0	109.6 ± 18.3*	151.2 ± 45.6	260.9 ± 101.6
Serum AST (IU/L)	Gal-3^+/+^	78.6 ± 7.6	80.7 ± 10.7	117.6 ± 27.3	75.7 ± 9.5
	Gal-3^−/−^	78.5 ± 11.5	114.9 ± 15.1	153.7 ± 26.6	192.1 ± 39.9*
Liver triglyceride (mg/g)	Gal-3^+/+^	13.3 ± 0.5	51.7 ± 3.4	64.9 ± 5.6	75.1 ± 1.7
	Gal-3^−/−^	15.7 ± 1.0	58.1 ± 4.1	66.0 ± 4.0	88.1 ± 10.7

Results are expressed as mean ± SEM of the values from 4–6 mice in each group. *P < 0.05 in comparison with gal3^+/+^ mice. CDAA: choline-deficient l-amino-acid-defined diet; ALT: alanine aminotransferase; AST: aspartate aminotransferase.

**Table 3:** Effect of CDAA diet and galectin-3 deficiency on the severity of hepatic steatosis and necroinflammation.

		CDAA diet consumption			
		0 wk	2 wk	4 wk	8 wk
Steatosis	Gal3^+/+^	0.0 ± 0.0	0.6 ± 0.5	1.5 ± 0.6	2.3 ± 1.0
	Gal3^−/−^	0.0 ± 0.0	2.0 ± 0.7*	2.8 ± 0.5*	2.8 ± 0.5
Necroinflammation	Gal3^+/+^	0.0 ± 0.0	0.6 ± 0.5	1.5 ± 0.6	2.0 ± 0.0
	Gal3^−/−^	0.0 ± 0.0	1.8 ± 0.4*	1.8 ± 0.5	2.0 ± 0.0

The severity of both hepatic steatosis and necroinflammation was scored on a scale of 0–3, as described in Materials and Methods. Results are expressed as mean ± SEM of the values from 4–6 mice in each group. *P < 0.05 in comparison with gal3^+/+^ mice.

**Table 4:** Genetic networks with high scores (>15) in gal3^−/−^ mice with CDAA diet-induced NAFLD.

Network	Genetic in Ingenuity networks^a^	Score	Focus genes	Top functions
1	ACTG1, ANXA2, AP3D1, CA3, CD36, CD151, CEACAM1, COTL1, CTSB, DNMT3A, ETS2, FOS, GAS1, GSTA5, GSTM3, HNRPAB, IFRD1, ITGA2B, JUN, MT1A, MT2A, MTF1, NMI, PBX2, RBM39, RBP1, RPL39, S100A10, SERPINA3G, SLPI, SPP1, TGM2, TMED10, TTRAP, TUBB2A	49	35	Cancer, cell death, cellular assembly and organization
2	BAG3, BMPR1A, C5ORF13, CD2AP, COL1A1, COL3A1, DIO1, DNAJB1, HDAC3, HSP90AA1, HSPA8, HSPA1A, HSPH1, IFIH1, IRF3, KLF6, KPNA4, LIFR, MBD1, P4HA1, PDGFC, PDGFRB, PPP1CB, PTPN2, RHOB, RND3, ROCK1, SERPINH1, STAT1, TANK, TBK1, TFRC, TGFBR2, TGTP, USP18	49	35	Cellular function and maintenance, dermatological diseases and conditions, organismal injury and abnormalities
3	ABCB11, ABCC3, BRD8, CDC20, CDH1, CPT1A, CSNK2A2, CYP3A5, CYP4A22, CYP7A1, DLG1, DNAJB4, DUSP6, FOXA1, G6PC, GADD45A, GADD45G, GCK, HNRPH2, IGHM, INSIG2, IQGAP1, LCN2, LGALS1, MAP3K4, PCK1, PPARGC1B, PTPRC, RXRA, SCD, SMAD4, SQLE, SSRP1, SUB1, TMSB4X	49	35	Carbohydrate metabolism, cell death, cancer
4	ACSL4, AFP, ALDOC, ARF6, ARMET, CES1, COL5A2, DUSP6, EGLN3, ENPP1, ERBB2, GJA1, HAS2, HGF, HIF1A, HRAS, LCN2, LDHA, LGALS1, NDST1, NID1, PDLIM1, PGAM1, PGF, PLOD1, PQLC1, PRDX2, RHOB, S100A4, SPRY2, SQLE, TMSB4X, TSC22D1, TSC22D4, UCRC	20	21	Cancer, cellular movement, reproductive system disease
5	ABCB1, ATF3, ATR, CASP4, CCNG1, CEBPD, CHUK, CSF1R, DUSP1, FOXO3A, GBP4, GPIAP1, GSTM5, H2AFZ, HSP90AA1, IFIT1L, ISG15, MCM2, MCM3, MCM4, MCM6, MDM2, NOX4, PDCD6IP, POLB, PPP2R4, RALGPS2, SON, SPP1, STRN3, SUMO1, TANK, TP53, UBQLN1, WT1	19	20	Cancer, cell cycle, reproductive system disease
6	ANXA2, CD14, CIITA, COL4A2, COL6A1, COL6A2, COL6A3, CREB1, ELN, EMILIN1, FGF10, GNAS, IRF7, LDHA, MAPKAPK2, MFI2, NCL, OAT, OPRM1, PCSK1, PITPNB, PLEKHC1, PTHLH, S100A11, SCD, SCOTIN, SEC63, SFTPB, SSTR2, TGFB1, TGM1, TH, TMEM123, TNFRSF10A, VNN1	17	19	Genetic disorder, skeletal and muscular disorders, dermatological diseases and conditions
7	ACVR1, AKAP12, CD14, CD53, CEACAM5, CEBPA, CPB2, CSF1R, CYP3A5, DDIT3, EPAS1, FCGR1A, GAL, HP, IGSF1, IK, IL6, INHBA, INHBC, INHBE, ITGA2B, LGALS4, LOX, PGD, PSCDBP, PTPRC, PTPRZ1, SAA1, SLCO1A1, SOAT1, SPBC25, STAR, TRIB1, UCP1, USP52	17	19	Lipid metabolism, molecular transport, small molecule biochemistry
8	ALDOB, APOM, ARPC4, ATP6V1E1, CD14, CD36, CEBPB, CPS1, CSF3, CSTB, CXCL6, CYP24A1, CYP4A22, CYP7A1, ELOVL3, FABP3, FGF19, HCA112, LCN2, LEP, MBOAT5, NFKBIZ, NRG1, ORM1, PDK4, PPARA, PTGER4, SCD, SLC10A2, SLC20A1, SRXN1, TNFSF11, TRAF6, UGP2, ZNF274	17	19	Carbohydrate metabolism, molecular transport, small molecule biochemistry

^a^Genes colored red or green are those identified by microarray analysis as upregulated and downregulated, respectively.
